# TLR2-Melatonin Feedback Loop Regulates the Activation of NLRP3 Inflammasome in Murine Allergic Airway Inflammation

**DOI:** 10.3389/fimmu.2020.00172

**Published:** 2020-02-07

**Authors:** Hui-Mei Wu, Cui-Cui Zhao, Qiu-Meng Xie, Juan Xu, Guang-He Fei

**Affiliations:** ^1^Department of Geriatric Respiratory and Critical Care, Anhui Geriatric Institute, The First Affiliated Hospital of Anhui Medical University, Hefei, China; ^2^Anhui Key Laboratory of Geriatric Molecular Medicine, Anhui Medical University, Hefei, China; ^3^Department of Respiratory and Critical Care, Anhui Geriatric Institute, The First Affiliated Hospital of Anhui Medical University, Hefei, China

**Keywords:** allergic airway inflammation, melatonin synthesis, NLRP3 inflammasome, TLR2, feedback loop

## Abstract

Toll-like receptor 2 (TLR2) is suggested to initiate the activation of NLRP3 inflammasome, and considered to be involved in asthma. The findings that melatonin modulates TLRs-mediated immune responses, together with the suppressing effect of TLRs on endogenous melatonin synthesis, support the possibility that a feedback loop exists between TLRs system and endogenous melatonin synthesis. To determine whether TLR2-melatonin feedback loop exists in allergic airway disease and regulates NLRP3 inflammasome activity, wild-type (WT) and TLR2^−/−^ mice were challenged with OVA to establish allergic airway disease model. Following OVA challenge, WT mice exhibited increased-expression of TLR2, activation of NLRP3 inflammasome and marked airway inflammation, which were all effectively inhibited in the TLR2^−/−^ mice, indicating that TLR2-NLRP3 mediated airway inflammation. Meanwhile, melatonin biosynthesis was reduced in OVA-challenged WT mice, while such reduction was notably rescued by TLR2 deficiency, suggesting that TLR2-NLRP3-mediated allergic airway inflammation was associated with decreased endogenous melatonin biosynthesis. Furthermore, addition of melatonin to OVA-challenged WT mice pronouncedly ameliorated airway inflammation, decreased TLR2 expression and NLRP3 inflammasome activation, further implying that melatonin in turn inhibited airway inflammation via suppressing TLR2-NLRP3 signal. Most interestingly, although melatonin receptor antagonist luzindole significantly reduced the protein expressions of ASMT, AANAT and subsequent level of melatonin in OVA-challenged TLR2^−/−^ mice, it exhibited null effect on leukocytes infiltration, Th2-cytokines production and NLRP3 activity. These results indicate that a TLR2-melatonin feedback loop regulates NLRP3 inflammasome activity in allergic airway inflammation, and melatonin may be a promising therapeutic medicine for airway inflammatory diseases such as asthma.

## Introduction

Asthma is one of the common respiratory diseases characterized by persistent or non-resolving allergen-driven inflammation. Toll-like receptors (TLRs), a member of pattern recognition receptors (PRRs), have been suggested to contribute to the initiation and perpetuation of airway inflammation, by virtue of their ability as the host's first line defense to recognize invading pathogens and aeroallergens (1). Within the TLRs family, TLR2 is regarded as the major one responsible for the sustaining airway inflammation and thus be most relevant to the onset of asthma (2–4). However, despite more and more advances in our understanding of the role of TLR2 in allergic asthma, the mechanism underlying TLR2 regulation in allergic airway inflammation is still elusive.

Recently, the other important type of intracellular PRRs, nucleotide-binding oligomerization domain-like receptors (NLRs) has been reported to be strongly associated with inflammatory diseases (5, 6). Particularly, as the best-characterized subtype shown to be expressed in airway, nucleotide-binding domain and leucine-rich repeat protein 3 (NLRP3) inflammasome is considered to be involved in the progress of asthma (7, 8). The NLRP3 inflammasome is a cytosolic protein complex composed of NLRP3, ASC, and pro-caspase-1. The activation of NLRP3 inflammasome proteolytically cleaves pro-caspase-1 into active caspase-1, which in turn promotes maturation of IL-1β and IL-18 (9). And these two NLRP3-associated cytokines are critical in the initiation and amplification of inflammatory process (5). Although NLRP3 inflammasome has been extensively investigated, its role in allergic airway inflammation is still controversial (10–14), and its regulatory networks remain elusive. TLRs and NLRs are two important kinds of PRRs, the formation and activation of NLRP3 inflammasome has been suggested to be initiated by TLRs (8, 15, 16). However, the crosstalk between TLR2 and NLRP3 inflammasome activity in the allergic airway diseases, as well as the underlying mechanism is not clear.

Melatonin (N-acetyl-5-methoxytrytamine), which is synthesized by the pineal gland or extra-pineal tissues (17), has been identified as a powerful immune-modulatory and anti-inflammatory molecule (18). Exogenous administration of melatonin has been reported to pronouncedly ameliorate airway inflammation (19, 20). Similarly, endogenous melatonin, which is produced from tryptophan (Trp) by converting 5-hydroxytryptamine (5-HT) successively by two key enzymes AANAT and ASMT, is also tightly associated with the pathogenesis of asthma (21, 22). It has been reported that endogenous melatonin synthesis is suppressed by activation of TLR9 (22), while other study shows that melatonin is able to inhibit TLRs-mediated inflammation (23), suggesting there may be a feedback loop between TLRs system and endogenous melatonin synthesis. More notably, it has been recently identified that a novel molecular target for melatonin is NLRP3 in murine model of septic response, liver injury and acute lung injury (24–26). However, it is uncertain whether TLR2-melatonin feedback loop exists in allergic airway disease, and regulates NLRP3 inflammasome activity.

To address these questions, we first examined whether TLR2 modulated melatonin biosynthesis and NLRP3 inflammasome activity by using TLR2^−/−^ mice, and further revealed whether this effect of TLR2 was feedback regulated by melatonin by administrating exogenous melatonin or melatonin receptor antagonist luzindole in OVA-induced allergic airway inflammation.

## Materials and Methods

### Mice

Female C57BL/6 mice were obtained from Shanghai Laboratory Animal Center, TLR2 deficient (TLR2^−/−^) mice on C57BL/6 background were presented by Dr. ZG. Tian (Institute of Immunology, School of Life Sciences, University of Science and Technology of China). All experimental protocols were approved by the Animal Care and Use Committee of Anhui Medical University.

### OVA-Induced Allergic Airway Inflammation and Interventions

The murine model of allergic airway inflammation was established according to previous study (27). Briefly, the mice (6–8 weeks old) were sensitized on day 0 with an intra-peritoneal injection of OVA (Sigma, St. Louis, MO, USA) emulsified with 1 mg potassium aluminum sulfate (Sangon Biotech, Shanghai, China) in 500 μl of saline. Airway inflammation was induced by inhalation of 1% aerosolized OVA 30 min per day, for consecutive 7 days. Control mice received saline-sensitization and inhalation of nebulized saline solution. The mice from WT-OVA-Melatonin group were administered with melatonin (10 mg/kg, Sigma), and the mice from TLR2^−/−^-OVA-Luzindole group were administered with luzindole (30 mg/kg, Sigma) 1 h before allergen-challenge through intra-peritoneal injection, respectively, the dose of melatonin and luzindole was used according to previous studies (19, 28). Experiments were performed with six mice per group. Mice were harvested 24 h following the last challenge.

### Bronchoalveolar Lavage Fluid (BALF) Collection and Cellular Analysis

The left lung bronchoalveolar lavage was performed with 500 μl ice-cold phosphate-buffered saline (PBS) for three times, and the resultant BALF was centrifuged to separate the cellular components. The cells were re-suspended in 200 μl PBS. Total cell counts were performed using a hemocytometer, differential cell counts were evaluated morphologically by staining cytospin slides of BALF samples with Wright Stain solution (Sigma).

### Lung Histopathological Examination

Immediately after collection of the BALF samples, lung tissues were fixed in 4% paraformaldehyde, and then embedded in paraffin before sagittally cutting into 5 μm sections. Subsequently, the sections were stained with hematoxylin/eosin (H&E) or periodic acid-Schiff (PAS) to analyze the inflammatory cells infiltration and global cell hyperplasia, respectively. Finally, a semi-quantitative method as described previously was used to evaluate the peribronchial inflammation and global cell hyperplasia (29, 30).

### Immunohistochemistry

Lung tissue sections (5 μM) were incubated with TLR2 antibody at 4°C overnight. Second day, the sections were incubated with HRP-conjugated secondary antibody (Beijing Golden Bridge Biotechnology, Beijing, China) for 1 h at 37°C. After rinsed for three times, sections were stained by diaminobenzidine (DAB) and counterstained by hematoxylin, and dark-brown staining was considered positive. The image were obtained under an Carl Zeiss Axio Scope.A1 (Carl Zeiss, German).

### Cytokines, 5-HT and Melatonin Determined by Enzyme-Linked Immunosorbent Assay (ELISA)

The level of OVA-specific IgE in serum was determined by ELISA using specific kits from Cusabio (Wuhan, China). The levels of IL-4, IL-13, IL-1β, IL-18, and 5-HT in BALF, the content of melatonin in lung homogenate were measured by ELISA kits from Cloud-Clone Crop (Wuhan, China) according to the protocols from the manufacturer.

### Western Blotting Analysis

Lung tissues were homogenized in RIPA in the presence of a protease inhibitor cocktail (Roche, Indianapolis, IN, USA) and Phosphatase Inhibitor Cocktail (Roche). The protein concentrations were determined by a BCA kit (Pierce, Rockford, IL, USA). Equal concentrations of protein (25 μg) were separated by SDS-PAGE on 12% acrylamide gels. The primary antibodies used were as follow: NLRP3 (Adipogen, San Diego, CA, USA), caspase 1 (p20) (Adipogen), IL-1β, pro-IL-1β (R&D Systems, Minneapolis, MN, USA), TLR2 (Millipore, Billerica, MA, USA), AANAT (Sigma), ASMT (Abcam, Boston, MA, USA) and GAPDH (KANGCHEN Biotech, Shanghai, China) for 1 h at 37°C, followed by at 4°C overnight. Blots were washed thrice with TBST and probed with appropriate secondary antibodies for 1 h at room temperature. After washing three times, the immune-reaction was analyzed using the ECL detection system (Pierce). The band density was determined by ImageJ software (NIH, Bethesda, MD, USA).

### Statistics

Results are showed as mean ± SEM. A one-way analysis of variance followed by Bonferroni multiple comparison test was used to determine the differences among the groups, except for analysis of TLR2 expression, which was performed using independent-sample *t*-test (SPSS 19.0). *P* < 0.05 was accepted significant.

## Results

### TLR2 Is Required for OVA-Induced Murine Allergic Airway Inflammation

**Figure 1:**
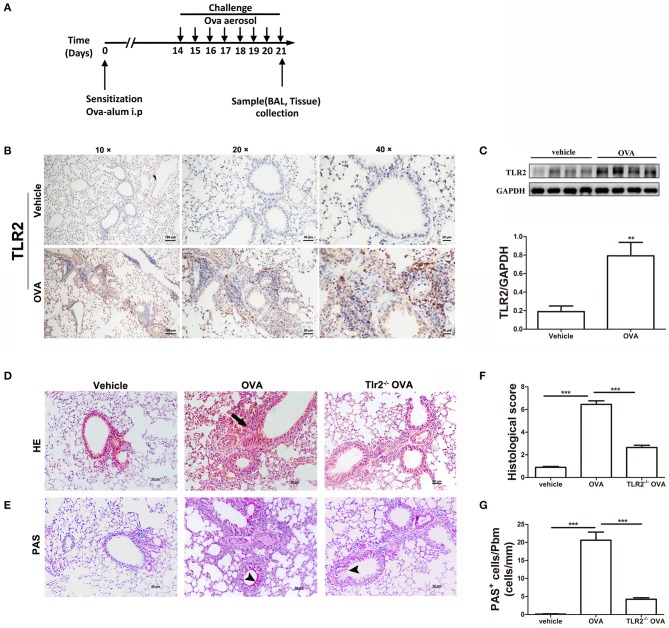
TLR2 is required for OVA-induced murine allergic airway inflammation. **(A)** Protocol of establishing allergic airway inflammation, and comparison of resolution of WT and TLR2^−/−^ mice. **(B)** The expression of TLR2 in lung tissue from vehicle and OVA-challenged mice was analyzed by immunohistochemistry. **(C)** The protein expression of TLR2 in OVA-challenged WT mice analyzed by western blot and quantification of the protein expression of TLR2. **(D)** Histological evaluation of the airway inflammation by staining lung sections with H&E, arrows indicates infiltrated leukocytes. **(E)** Histological examination of mucus production in the lung sections stained with PAS, arrow heads indicates goblet cells. **(F)** Quantitative analysis of airway inflammation. **(G)** Quantitative of mucus production. Scale bar: 50 μm. **p < 0.01, ***p < 0.001.

We first set out to confirm the role of TLR2 in murine allergic airway disease. WT and TLR2^−/−^ mice were sensitized and challenged with OVA following the protocol showed in Figure 1A. Immunohistochemistry and western blot results showed that TLR2 protein expression was significantly increased in OVA-challenged WT mice in comparison with that of control mice (Figures 1B,C), and TLR2 was expressed on various types of cells, such as epithelial cells and leukocytes. Concomitantly, lung histology showed an increase in leukocyte recruitment to peribronchial and mucous cell metaplasia in OVA-challenged WT mice (Figures 1D–G). In sharp contrast, OVA-challenged TLR2^−/−^ mice showed reductions in inflammatory cells recruitment (Figures 1D,F) and airway PAS^+^ cells (Figures 1E,G) in comparison with that of OVA-challenged WT mice.

### TLR2 Is Required for OVA-Induced Inflammatory Cells Infiltration, IgE, and Th2 Cytokines Production

**Figure 2:**
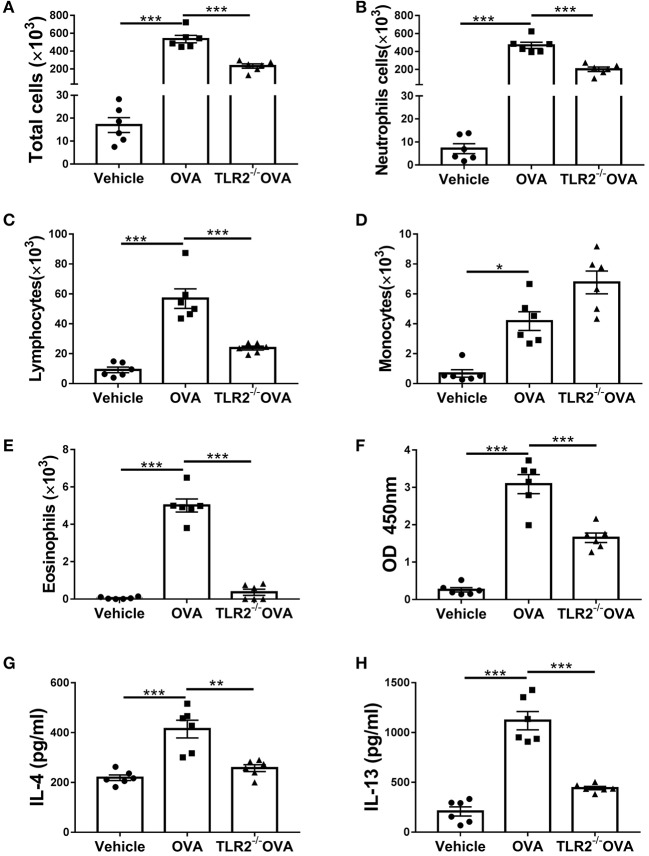
TLR2 is required for OVA-induced inflammatory cells infiltration, IgE and Th2 cytokines production. **(A)** Total cell counts in the BALF of WT and TLR2^−/−^ mice. **(B–E)** Differential cell counts in BALF of WT and TLR2^−/−^ mice. **(F)** The level of OVA-specific IgE in serum. **(G,H)** Productions of IL-4 and IL-13 in BALF of WT and TLR2^−/−^ mice were analyzed by ELISA. *p < 0.05, **p < 0.01, ***p < 0.001.

In addition to lung histological changes, airway challenged with OVA induced a significant increase in total BALF cellularity in comparison with that of control mice (Figure 2A). Further morphologic assessments of differentially stained BALF samples revealed that the increase in cellularity was resulted from a significant influx of neutrophils, lymphocytes, monocytes and eosinophils (Figures 2B–E). However, in comparison with WT mice, the total number or composition of the BALF cellularity in TLR2^−/−^ mice post OVA challenge was significantly decreased except for monocytes, which trended to increase but did not reach statistical significance (Figures 2A–E). Meanwhile, the level of OVA-specific IgE in TLR2^−/−^ mice was significantly lower than that of WT mice (Figure 2F). Furthermore, significant increase in the levels of Th2-associated cytokines including IL-4 and IL-13 was observed in OVA-challenge WT mice (Figures 2E,F). Similarly, significant differences between WT and TLR2^−/−^ mice were observed that TLR2 deficiency significantly decreased the levels of these two Th2-associated cytokines post OVA challenge (Figures 2G,H). Together, these data supported the role of TLR2 in the development of allergic airway inflammation in this OVA model.

### OVA-Induced Activation of NLRP3 Inflammasome and Decrease of Melatonin Biosynthesis Are TLR2 Dependent

**Figure 3:**
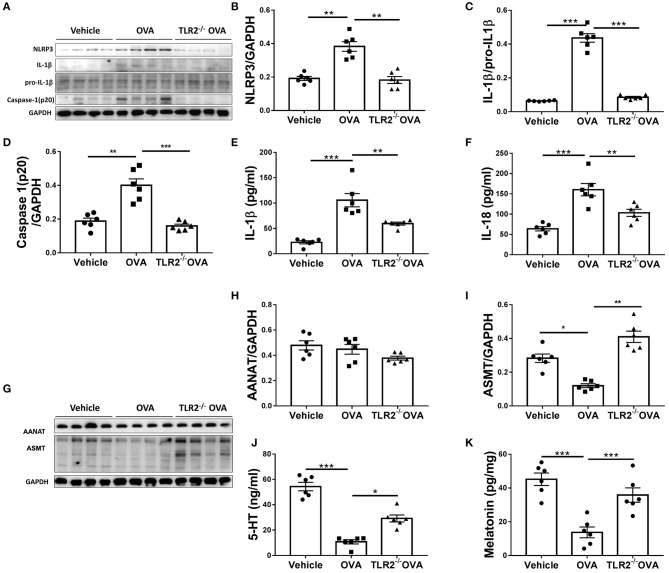
OVA-induced activation of NLRP3 inflammasome and decrease of melatonin biosynthesis are TLR2 dependent. **(A)** NLRP3, mature IL-1β, pro-IL-1β, and caspase1(p20) western blot analysis. **(B–D)** Relative ratio of NLRP3 to GAPDH, IL-1β to pro-IL-1β, and caspase1(p20) to GAPDH. **(E,F)** ELISA analysis of IL-1β and IL-18 in BALF. **(G)** Western blot analysis of AANAT and ASMT in lung tissues. **(H,I)** Relative ratio of AANAT or ASMT to GAPDH. **(J,K)** The levels of 5-HT in BALF and melatonin in the lung homogenate were analyzed by ELISA. *p < 0.05, **p < 0.01, ***p < 0.001.

We next questioned how TLR2 regulated allergic airway inflammation. It has been shown that NLRP3 inflammasome is associated with allergic airway disease in response to OVA (10). We next assessed the link between TLR2 and NLRP3 inflammasome activity. Our results showed that NLRP3, cleaved form of IL-1β and caspase 1(p20) were increased in OVA-challenged WT mice in comparison with those of control mice, while such increase was completely abrogated in TLR2^−/−^ mice following OVA challenge (Figures 3A–D). Similarly, productions of NLRP3-associated IL-1β and IL-18 were markedly decreased in OVA-challenged TLR2^−/−^ mice, comparable to those of control mice (Figures 3E,F). Taken together, in this OVA model, NLRP3 inflammasome activated by OVA required licensing through TLR2, suggesting that TLR2-NLRP3 axis mediated OVA-allergic airway inflammation.

Consequently, we sought to investigate the regulatory networks how TLR2-NLRP3 axis mediated allergic airway diseases. Previous study has shown that TLR9 negatively regulates melatonin production in response to OVA challenge, and this endogenous synthesized melatonin may regulate airway inflammation (22). Here, our present study showed that OVA notably suppressed the protein expression of ASMT but not AANAT in lung tissues (Figures 3G–I), and lowered the level of 5-HT in BALF and melatonin in lung homogenate in WT mice (Figures 3J,K), while these reductions were significantly restored by TLR2 deficiency (Figures 3H–K). These data confirmed that besides TLR9, TLR2, another member of TLRs family suppressed endogenous melatonin biosynthesis in OVA-induced allergic airway inflammation, therefore suggesting that TLR2-NLRP3 -mediated allergic airway inflammation was associated with decreased endogenous melatonin biosynthesis.

### The Effect of Melatonin or Luzindole on OVA-Induced Airway Inflammation

**Figure 4:**
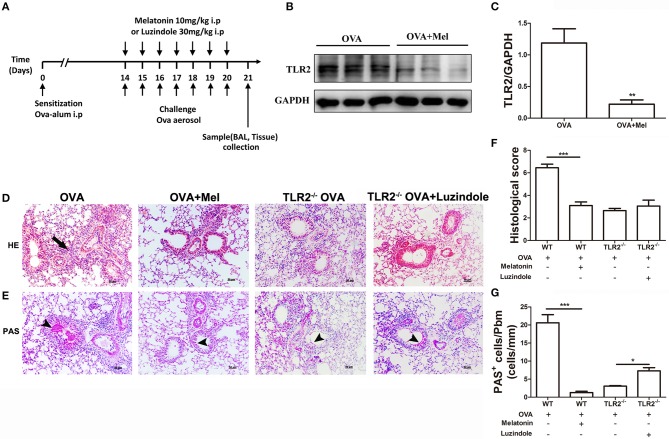
The effect of melatonin or luzindole on OVA-induced airway inflammation. **(A)** Protocol of administration of melatonin or luzindole during establishing allergic airway diseases model. **(B,C)** Protein expression of TLR2 in lung tissues of OVA-challenged WT mice treated with melatonin or not. **(D)** Lung histopathology based on H&E staining to evaluate inflammatory cell infiltration, arrows indicates infiltrated leukocytes. **(E)** Mucus secretion was analyzed based on PAS staining, arrow heads indicates goblet cells. **(F)** Histological scoring of lung inflammation. **(G)** Quantification of mucus secretion. *p < 0.05, **p < 0.01, ***p < 0.001.

Another important question arises whether endogenous melatonin increased by TLR2 deficiency directly suppresses NLRP3 inflammasome activity or feedback controls TLR2-NLRP3 signal. To address this question, melatonin or its receptor antagonist luzindole was applied as illustrated in Figure 4A. First, we found that administration of melatonin significantly attenuated the protein level of OVA-induced TLR2 in WT mice (Figures 4B,C). Meanwhile, melatonin-treated mice showed reductions in leukocyte recruitment and mucus productions compared with vehicle-treated WT mice post OVA challenge (Figures 4D–G). However, administration of luzindole only significantly promoted mucus productions in comparison with vehicle-treated TLR2^−/−^ mice post OVA challenge (Figures 4D–G).

### The Effect of Exogenous Melatonin or Luzindole on OVA-Induced Inflammatory Cells Infiltration, IgE, and Th2 Cytokines Production

**Figure 5:**
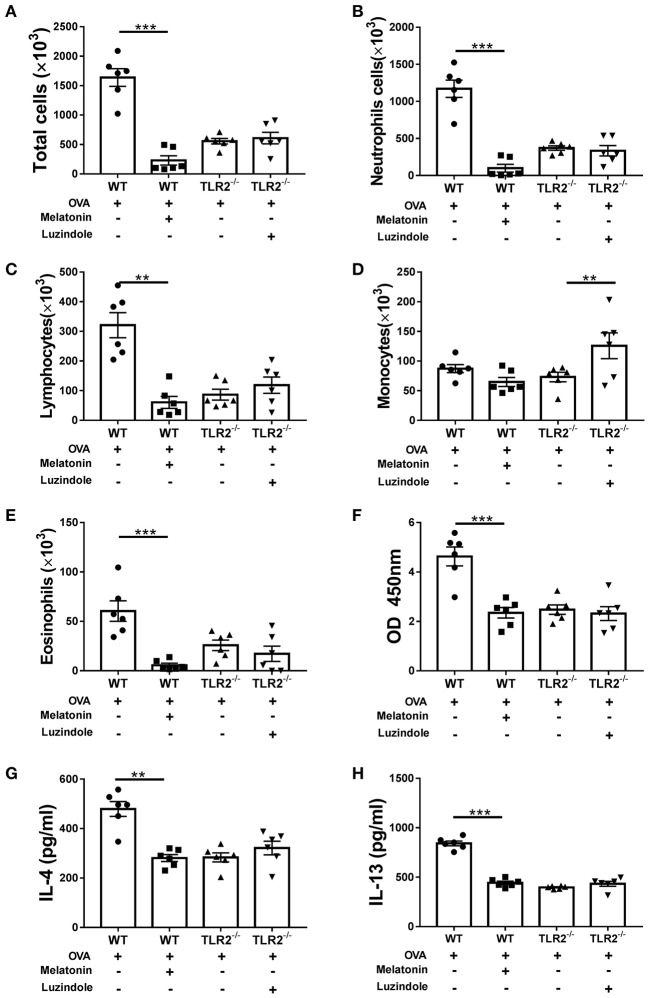
The effect of exogenous melatonin or luzindole on OVA-induced inflammatory cells infiltration, IgE and Th2 cytokines production. **(A–E)** Analysis of total and differential cells found in BALF of OVA-challenged WT or TLR2^−/−^ mice after melatonin or luzindole treatment, respectively. **(F)** The level of OVA-specific IgE in serum. **(G,H)** The levels of IL-4 and IL-13 in BALF. **p < 0.01, ***p < 0.001.

Moreover, upon OVA challenge, melatonin treatment dramatically reduced the total number or composition of the BALF cellularity except for monocytes (Figures 5A–E), lowered the concentration of IgE in serum (Figure 5F), and decreased the productions of IL-4 and IL-13 in BALF (Figures 5G,H) in comparison with those in vehicle-treated WT mice. Interestingly, no significant difference was found between luzindole-treated and vehicle-treated TLR2^−/−^ mice in BALF cellularity or production of IgE and Th2-associated cytokines following OVA challenge (Figures 5A–C,E,F). We only found significant increase in monocytes infiltration in luzindole-treated TLR2^−/−^ mice (Figure 5D). These results demonstrated that melatonin remarkably promoted resolution of allergic airway inflammation by down-regulating TLR2 signaling. However, blocking the effect of melatonin in TLR2^−/−^ mice by luzindole only aggravated allergen-induced mucus hyper-secretion, but had null effect on leukocyte infiltration and Th2 cytokines production.

### The Effect of Exogenous Melatonin or Luzindole on NLRP3 Inflammasome Activation and Endogenous Melatonin Synthesis

**Figure 6:**
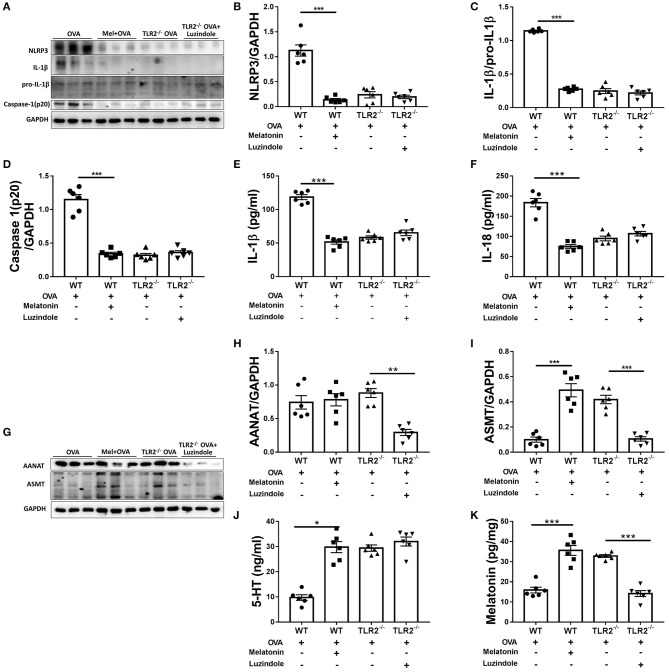
The effect of exogenous melatonin or luzindole on NLRP3 inflammasome activation and endogenous melatonin synthesis. **(A)** Representative blots showing the protein expression of NLRP3, mature IL-1β, pro-IL-1β, and caspase1(p20) in lung homogenate. **(B–D)** Relative band densities of NLRP3 and caspase1(p20) were analyzed by normalizing to GAPDH, relative band densities of IL-1β was analyzed by normalizing to pro-IL-1β. **(E,F)** The levels of IL-1β, IL-18 in BALF were detected by ELISA. **(G)** Representative blots showing the levels of AANAT and ASMT in lung homogenate. **(H,I)** Relative band densities of AANAT and ASMT were analyzed by normalizing to GAPDH. **(J,K)** The level of 5-HT in BALF and melatonin in the lung homogenate were detected by ELISA. *p < 0.05, **p < 0.01, ***p < 0.001.

Subsequently, we showed that administration of melatonin dramatically decreased the protein expressions of NLRP3, mature IL-1β and caspase 1(p20), lowered the productions of IL-1β and IL-18 in comparison with those in vehicle-treated WT mice following OVA challenge (Figures 6A–F). Importantly, NLRP3 inflammasome activity was not significantly different between vehicle-treated and luzindole-treated TLR2^−/−^ mice post OVA challenge (Figures 6A–F). These data suggested that melatonin elicited its effect on NLRP3 inflammasome activity via TLR2 signaling, when TLR2 was deficient, blocking the effect of melatonin had null effect on NLRP3 inflammasome activity.

Finally, we found that treatment of melatonin resulted in dramatic increase in the protein expression of ASMT, marked elevation in the levels of 5-HT and melatonin, while did not affect the AANAT expression in comparing with vehicle-treated WT mice (Figures 6G–K). The protein expression of ASMT in lung tissues, the levels of 5-HT in BALF and melatonin in lung homogenate in melatonin-treated WT mice were comparable to that in vehicle-treated TLR2^−/−^ mice post OVA challenge. However, TLR2^−/−^ mice treated with luzindole showed notably reduced expressions of both AANAT and ASMT (Figures 6G–I), and lowered the level of melatonin in lung homogenate but not 5-HT in BALF in comparison with that of vehicle-treated TLR2^−/−^ mice following OVA challenge (Figures 6J,K). Therefore, our study suggested that administration of melatonin further promoted endogenous melatonin biosynthesis, while treatment of luzindole significant inhibited endogenous melatonin synthesis in TLR2^−/−^ mice.

## Discussion

In the present study, we demonstrated that OVA-induced allergic airway inflammation occurred in parallel with increased-expression of TLR2, activation of NLRP3 inflammasome and decreased biosynthesis of melatonin. Deletion of TLR2 effectively alleviated allergic airway inflammation and concomitantly inhibited the activation of NLRP3 inflammasome as well as restored the level of melatonin. Furthermore, exogenous addition of melatonin to OVA-challenged WT mice pronouncedly ameliorated airway inflammation, decreased OVA-induced TLR2 expression and NLRP3 activity, and increased melatonin biosynthesis to similar level as that in OVA-challenged TLR2^−/−^ mice. However, although luzindole significantly reduced the expression of AANAT and ASMT and subsequent level of melatonin in OVA-challenged TLR2^−/−^ mice, it exhibited null effect on OVA-induced airway inflammation and activation of NLRP3, it only aggravated allergen-induced mucus hyper-secretion. These results are the first to show the existence of TLR2-melatonin feedback loop in allergic airway diseases, which regulates NLRP3 inflammasome activity and may represent a mechanism underlying the initiation and persistence of airway inflammation.

An allergic airway disease is usually dominated by a strong Th2 response which can be redirected to Th1 response by regulation of TLRs signaling (4, 31, 32), making TLRs attractive therapeutic targets. During the last decade, accumulating evidence has been focused on the role of TLRs in the pathogenesis of airway inflammatory diseases such as asthma and the possibility of using TLRs-based therapies for asthma (33, 34). To date, TLR2 has been considered as the most relevant to the onset of asthma. Asthmatic patients who ultimately die have increased expression of TLR2 (2), activation of TLR2 promotes Th2-biased immune responses, which may be correlated with the imbalance of Th1/Th2 in asthma (3, 4). In line with these data, our present study demonstrated a significant increase of TLR2 expression in WT mice post OVA challenge, and this increase was accounted from its induced-expression on various cell types. Previous studies also indicate its wide spectral expression (35–37), and reports from our group and others have indicated that TLR2 on macrophages (38), group 2 innate lymphoid cells (39) and epithelial cells (40) may contribute to allergic airway inflammation. Furthermore, the increased expression of TLR2 was accompanied with lung inflammation exacerbation in WT mice post OVA challenge in this study. However, such OVA-induced airway inflammation, including leukocyte recruitment to bronchial, mucus metaplasia in the airways, total number or composition of the BALF cellularity, the level of OVA-specific IgE, as well as IL-4 and IL-13, was notably alleviated in OVA-challenged TLR2^−/−^ mice. Based on these data, TLR2 was considered to mediate allergic airway inflammation, and targeting TLR2 may have therapeutic benefit in allergic airway diseases.

However, the mechanism underlying TLR2 mediation of allergic airway inflammation is still unknown. NLRP3 inflammasome is one of the most extensively characterized NLRs due to its relevance in human inflammatory disorders such as asthma. Invading pathogens including viral or bacterial which commonly associated with asthma exacerbation, have been shown to trigger NLRP3 activation (5, 41). IL-1β is increased in the serum and BALF of human asthmatics, and administration of IL-1β induces airway hyper-reactivity (12, 13). Increased expressions of NLRP3, caspase-1 and IL-1β are found in macrophages as well as neutrophils in sputum of neutrophilic asthma (14). Consistent with these studies, our present study showed that in WT mice, OVA challenge substantially increased protein expression of NLRP3, mature IL-1β and caspase 1(p20) in lung tissues, markedly elevated the levels of NLRP3-associated IL-1β and IL-18 in BALF. However, this effect was only seen in WT mice, but not in TLR2^−/−^ mice. The interaction between TLR2 and NLRP3 inflammasome has been also reported in various cell types. In monocytes (42), macrophages (43, 44), bone marrow-derived DCs (45) and several different cell lines (46, 47), lacking TLR2 failed to upregulate NLRP3 inflammasome as well as its substrate IL-1β. Therefore, our results have suggested that activation of NLRP3 inflammasome induced by OVA requires TLR2 signaling, thus TLR2 may mediate allergic airway inflammation through regulating NLRP3 inflammasome activity.

A question arises now is, by which TLR2 crosstalks with NLRP3 inflammasome and consequently mediates allergic airway inflammation. Melatonin has been reported to exert important immune-modulating effects in allergic airway diseases (19, 20, 48, 49), and the level of melatonin in saliva or serum of asthma patients were significantly lower than those in healthy controls (21, 50, 51). Particularly, it has been shown that TLR9 signaling regulates endogenous melatonin synthesis in allergic airway inflammation (22). Considering TLR2 and TLR9 are structurally similar, we asked whether TLR2 regulates endogenous melatonin synthesis and thereby modulated NLRP3 inflammasome activity. Indeed, the current study demonstrated that TLR2 protein expression was remarkably increased accompanying with decreased expression of melatonin synthetase ASMT and lower level of melatonin in lung homogenate in WT mice post OVA challenge, while deletion of TLR2 significantly rescued melatonin biosynthesis. Since it have been reported that C57BL/6 mice were pineal melatonin-deficient mice (52, 53) or produce much lower melatonin in their pineal gland (17, 54), suggested an extra-pineal melatonin synthesis might compensate for the pineal deficiency (17). Actually, there are results reporting that several extra-pineal tissues, such as thymus, spleen and skin, in C57BL/6 mice can synthesize melatonin (17, 55), our current study further found that another extra-pineal tissue, i.e., lung synthesized melatonin, and this process maybe modulated by TLR2 signal.

On the other hand, melatonin has been evidenced to significantly inhibit airway inflammation (49, 56), and suppress TLR3/4-mediated inflammation in liver injury (23). Most notably, NLRP3 is a novel molecular target for melatonin in murine model of septic response, liver injury and acute lung injury (24–26). Consistent with these findings in animals, melatonin also has been shown to exert inhibitory effect on TLRs including TLR3/4/9 signaling in macrophage (57–59) and NLRP3 inflammasome in epithelial cells (60). Therefore, we asked whether endogenous melatonin, which was regulated by TLR2 signal, alleviated allergic airway inflammation through directly suppressing NLRP3 inflammasome activity or feedback controlling TLR2-NLRP3 signal. Our current study showed that treatment of melatonin notably alleviated OVA-induced airway inflammation in WT mice, which was consistent with previous findings (19, 56). However, we extended previous observations by showing that melatonin treatment strongly inhibited OVA-induced protein expressions of TLR2, NLRP3, mature IL-1β and caspase1(p20), as well as lowered the levels of NLRP3-associated IL-1β and IL-18 in BALF in WT mice, suggesting that melatonin mitigates allergic airway inflammation by inhibiting TLR2 and NLRP3 inflammasome activity. Considering the data shown above that TLR2 signaling regulated melatonin synthesis, we speculated that a TLR2-melatonin feedback loop may exist in allergic airway disease, and melatonin elicits its effect on activation of NLRP3 inflammasome through TLR2 signal. Additionally, we found that exogenous addition of melatonin further increased protein expression of ASMT, as well as elevated the level of 5-HT in BALF and melatonin in lung homogenate in OVA-challenged WT mice. These interesting data suggested that the proven effect of exogenous melatonin in the resolution of inflammation was paralleled by the effect of endogenous synthesized melatonin.

Among the three melatonin receptors, the anti-inflammatory effect of melatonin is reported to be mainly mediated by MT2 melatonin receptor (61). Luzindole is considered as a non-selective MT1/MT2 receptor antagonist, showing 15–26 times higher affinity for MT2 receptor, which pharmacologically blocks the effect of melatonin (62). Therefore, luzindole was used as indicated (28) to further confirm that melatonin elicited its effect on NLRP3 inflammasome activity via TLR2 signal. Our results showed that although administration of luzindole to OVA-challenged TLR2^−/−^ mice significantly reduced the expression of melatonin synthetase AANAT and ASMT, and subsequent level of melatonin, it exhibited null effects on the level of 5-HT in BALF, OVA-induced leukocyte infiltration, the level of IgE, Th2 cytokines production, and NLRP3 inflammasome activity, it only aggravated allergen-induced mucus hyper-secretion. To our knowledge, this is the first study showing that luzindole inhibited lung melatonin synthesis, which may be due to the blockage of 5-HT conversion to melatonin. These combined data suggested that the effect of melatonin on airway inflammation and NLRP3 inflammasome activity requires TLR2 signaling, and melatonin which is modulated by TLR2 signal feedback regulates TLR2 signaling, and subsequently regulates NLRP3 inflammasome activity and airway inflammation. However, the inhibitory effect of melatonin on airway remodeling characterized by mucus hyper-secretion may not require TLR2 signal, because luzindole still deteriorates mucus production even in the absence of TLR2 signal.

**Figure 7:**
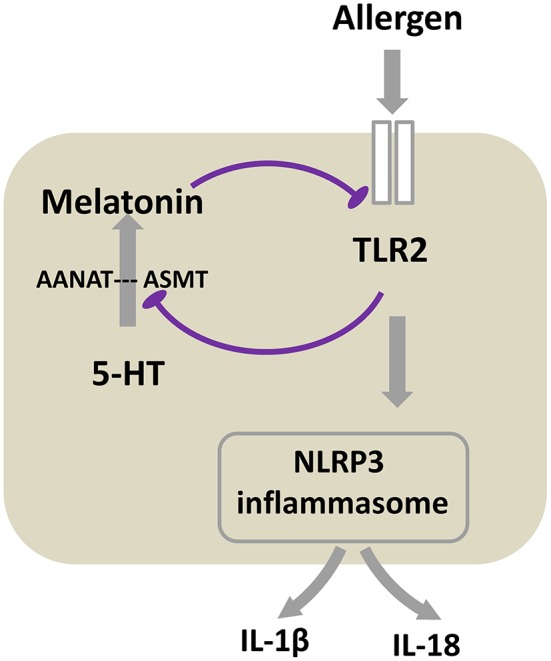
Proposed schematic of the mechanism that TLR2-melatonin feedback loop regulates the activation of NLRP3 inflammasome in allergic airway inflammation. After TLR2 is activated by allergen, it subsequently induces NLRP3 inflammasome activation. Meanwhile, TLR2 inhibits the expression of melatonin synthetase ASMT, accordingly blocks 5-HT converting to melatonin, ultimately leading to the decreased level of melatonin. This inhibition of melatonin synthesis results in the loss of its inhibitory effect on the TLR2 signaling. The result of the cycle is the persistent of allergic airway inflammation.

In conclusion, the present study demonstrates that a TLR2-melatonin feedback loop regulates the activation of NLRP3 inflammasome in allergic airway inflammation, and suppression of melatonin synthesis by TLR2 activation in turn results in the loss of its inhibitory effect on the TLR2 signaling (Figure 7). This important endogenous regulatory feedback loop may drive the onset of allergic airway inflammation, and melatonin may be a promising therapeutic medicine for airway inflammatory disease such as asthma. The present study used OVA model, which has been the most widely used pre-clinical allergic asthma model and recapitulates many of the hallmarks of allergic asthma in humans, however, bio-models offer other allergen-induced allergic model, such as house dust mite (HDM), *Alternaria*, papain and IL-33, with each mimicking the major features of human asthma. When OVA, HDM, or *Alternaria* was given via inhalation or intranasally, each of them can be recognized and sensed by TLRs reportedly, especially TLR2 (40). Similarly, intranasally given IL-33 is reported can induce direct stimulation of TLR2 on ILC2s (39). However, papain, which is homologous of HDM-derived Der p1, activates innate immune responses in a manner distinct from that of PAPMs and can induce production of an alarmin IL-33 (63). We hypothesize our findings may be also applicable to other allergens–induced allergic airway inflammation models in which TLR2 is activated by allergen. However, the true fact needs future study to discover.

## Data Availability Statement

The raw data supporting the conclusions of this article will be made available by the authors, without undue reservation, to any qualified researcher.

## Ethics Statement

This study was carried out in accordance with the recommendations outlined in Animal Care and Use Committee guideline of Anhui Medical University. The protocol was approved by the Animal Care and Use Committee guideline of Anhui Medical University.

## Author Contributions

H-MW designed and carried out most of the experiments, analyzed the data, and wrote the manuscript. C-CZ, Q-MX, and JX established the murine model of allergic airway disease, done western blotting, and analyzed the data. G-HF designed the experiment and revised the manuscript.

### Conflict of Interest

The authors declare that the research was conducted in the absence of any commercial or financial relationships that could be construed as a potential conflict of interest.
